# A Case of Drug-Induced Cystitis Associated With a Multicomponent Herbal Formulation Involving Yokuinin-To

**DOI:** 10.7759/cureus.110922

**Published:** 2026-06-15

**Authors:** Taro Bando, Kazunobu Aramaki, Yuto Tsubonuma, Seiya Shiramizu, Naohiro Fujimoto

**Affiliations:** 1 Urology, Local Incorporated Administrative Agency Kurate Hospital, Kurate, JPN; 2 Urology, University of Occupational and Environmental Health, Kitakyushu, JPN; 3 Urology, Kyushu Rosai Hospital Moji Medical Center, Kitakyushu, JPN

**Keywords:** adverse event, drug-induced cystitis, herbal medicine, kampo, yokuinin-to

## Abstract

Herbal medicines, including traditional Japanese Kampo formulations, are widely used for various conditions. Although generally considered safe, they may cause unexpected adverse events, which are often underrecognized.

We report a case of cystitis potentially associated with a traditional Japanese multicomponent herbal formulation. A 77-year-old man presented with dysuria, urinary frequency, and gross hematuria. The patient had been taking a Kampo formulation for 35 days. Laboratory studies showed hematuria without evidence of a urinary tract infection. Computed tomography and cystoscopy revealed bladder wall thickening with erythematous and edematous changes.

Given the temporal relationship between initiation of the herbal formulation and symptom onset, drug-induced cystitis was suspected. Discontinuation of the formulation led to marked improvement in symptoms, urinalysis findings, and imaging results without additional specific treatment.

Bladder malignancy and interstitial cystitis were considered unlikely based on cystoscopic findings, imaging studies, urine cytology, and spontaneous resolution of the lesions, although eosinophilic and viral cystitis could not be completely excluded.

## Introduction

Herbal medicines are widely used worldwide for a variety of conditions. Kampo medicine, a traditional Japanese herbal medicine system derived from traditional Chinese medicine, has been integrated into modern Japanese healthcare and is routinely used in daily clinical practice in Japan. It comprises numerous formulations used for diverse conditions, and specific formulations are selected according to the patient's symptoms and clinical condition.

Although they are often perceived as safe, accumulating evidence suggests that they are not free of adverse effects, and such events may be underrecognized in clinical practice because of underreporting and variability in reporting systems.

Although most cases of cystitis are caused by bacterial infection, various medications can induce cystitis [1]; although rare, herbal medicines, including traditional Japanese Kampo formulations, have also been reported to cause cystitis [2].

Here, we report a case of cystitis potentially associated with a traditional Japanese multicomponent herbal formulation (Kampo), which may represent the first reported case involving Yokuinin-to. Previous reports have indicated that herbal medicines containing components of Yokuinin-to may cause cystitis [2]. However, to our knowledge, Yokuinin-to-associated cystitis has not been previously reported.

## Case presentation

A 77-year-old man was prescribed Yokuinin-to (Tsumura & Co., Tokyo, Japan; 7.5 g/day, administered in three divided doses), a Kampo medicine, by an orthopedist for bilateral shoulder pain because it is indicated for arthralgia and myalgia [3]. He had no history of urological disease. Approximately 20 days after initiating the medication, he developed decreased voided volume (approximately 150 mL per void) and urinary frequency (>10 times per day, with three episodes of nocturia). A few days later, dysuria developed.

Although Yokuinin-to was discontinued after 35 days of treatment, the patient developed gross hematuria four days later and was subsequently referred to our department.

His past medical history included surgery for spondylolysis, bilateral rotator cuff repair, gastric ulcer, and herpes zoster. He had a history of allergies to beef, pork, natto (fermented soybeans), green and yellow vegetables, and synthetic fibers. His medications included acetaminophen and acetylcysteine hydrochloride.

Clinical laboratory findings at the initial presentation are summarized in Table 1. Urinalysis revealed >100 red blood cells per high-power field, 1-4 white blood cells per high-power field, and no bacteria. Urine cytology was class II (negative for malignancy). Blood test results were unremarkable.

**Table 1:** Clinical laboratory findings at the initial presentation. WBC: white blood cell count, RBC: red blood cell count, Hb: haemoglobin, Plt: platelet count, TP: total protein, ALB: albumin, AST: aspartate aminotransferase, ALT: alanine aminotransferase, Na: sodium, K: potassium, Cl: chloride, Ca: calcium, BUN: blood urea nitrogen, Cre: creatinine, eGFR: estimated glomerular filtration rate, pH: potential of hydrogen, HPF: high-power field.

Investigation	Result	Reference range
Hematology		
WBC (×10³/μL)	5.51	3.30–8.60
Basophils (%)	0.4	0–1.0
Eosinophils (%)	4.7	0–6.0
Neutrophils (%)	58.6	37.0–72.0
Lymphocytes (%)	27.4	18–59
Monocytes (%)	8.9	0–12
RBC (×10⁶/μL)	4.42	4.35–5.55
Hb (g/dL)	14.5	13.7–16.8
Plt (×10³/μL)	152	158–348
Biochemistry		
TP (g/dL)	6.9	6.6–8.1
ALB (g/dL)	3.8	4.1–5.1
AST (U/L)	33	13–30
ALT (U/L)	27	10–42
Na (mmol/L)	139	138–145
K (mmol/L)	4.5	3.6–4.8
CL (mmol/L)	104	101–108
Ca (mg/dL)	9.0	8.8–10.1
BUN (mg/dL)	26	8.0–20
Cre (mg/dL)	0.97	0.65–1.07
eGFR (mL/min/1.73 m²)	57.7	-
Urinalysis		
Protein	2＋	Negative
Glucose	Negative	Negative
Occult blood	3＋	Negative
Urobilinogen	Normal	Normal
Ketone bodies	Negative	Negative
pH	6	5.0–8.0
Specific gravity	1.03	1.002–1.03
Red blood cells	>100/HPF	0–4/HPF
White blood cells	0–4/HPF	0–4/HPF
Bacteria	Negative	Negative

Computed tomography showed marked thickening of the anterior wall to the dome of the bladder (Figure 1a,b). Cystoscopy revealed prominent edema and multiple hemorrhagic lesions in the corresponding region (Figure 2).

**Figure 1:**
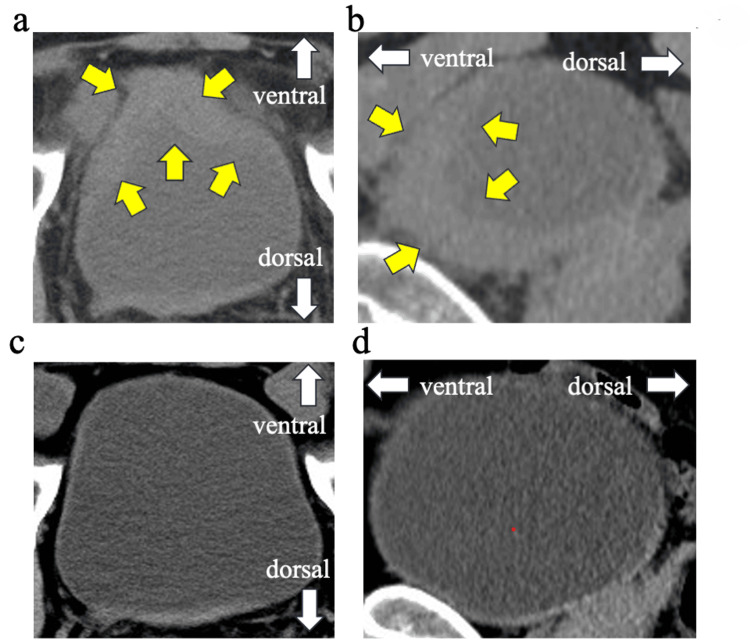
Computed tomography findings. Axial (a) and sagittal (b) images obtained at the initial presentation show marked thickening of the anterior bladder wall extending to the dome. Axial (c) and sagittal (d) images obtained three months after discontinuation of Yokuinin-to demonstrate complete resolution of the bladder wall thickening.

**Figure 2:**
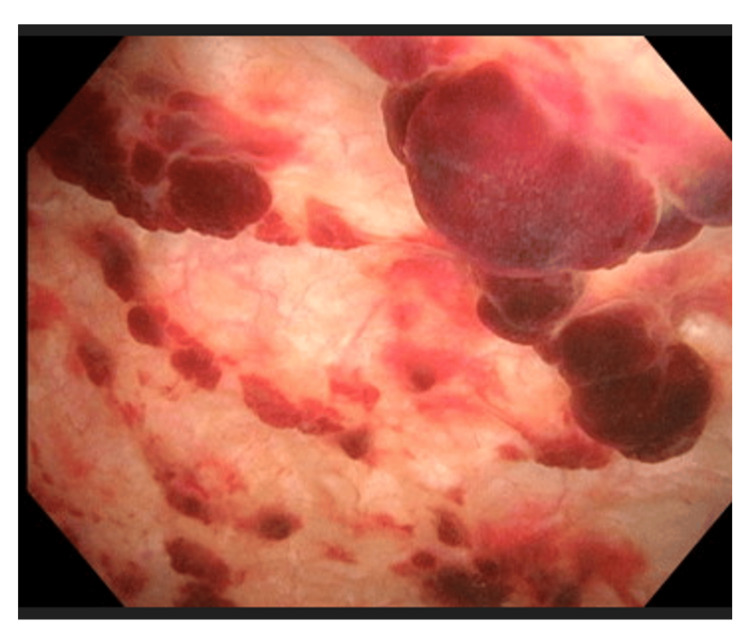
Cystoscopy of the anterior bladder wall reveals marked edema and multiple hemorrhagic lesions, consistent with inflammatory changes.

Nonbacterial cystitis was suspected, and no specific treatment was initiated. The patient's symptoms gradually improved and resolved approximately 50 days after discontinuation of Yokuinin-to, and the urinalysis findings normalized (Figure 3).

**Figure 3:**
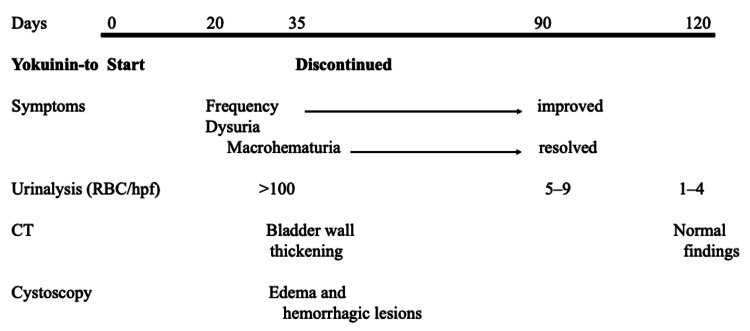
Clinical course. Timeline of symptom progression, urinalysis findings, imaging findings, and cystoscopic findings before and after discontinuation of Yokuinin-to. CT: computed tomography, RBC: red blood cell, hpf: high power field.

Follow-up computed tomography performed three months after discontinuation of Yokuinin-to showed complete resolution of the bladder wall thickening (Figure 1c,d).

## Discussion

Drug-induced cystitis (DIC) can be caused by a variety of agents, including chemotherapeutic drugs [1], nonsteroidal anti-inflammatory drugs [1,4], antiallergic agents such as tranilast [5], antibiotics [6,7], and herbal medicines, including Kampo formulations [2].

The underlying mechanisms of DIC are thought to involve direct injury to the bladder mucosa and immunologic reactions to the drug. Cyclophosphamide is a well-known agent that causes bladder mucosal injury [1]. In contrast, drugs such as tranilast [5] and penicillin [6,7] have been reported to induce DIC via hypersensitivity reactions.

DIC associated with herbal medicines is also considered to be related to allergic mechanisms, and the patient's predisposition to multiple allergies may have contributed to the development of cystitis in this case.

Previous reports have described various immunoallergic adverse events associated with Kampo medicines, including liver injury, interstitial pneumonia, allergic cystitis, and drug eruptions [8]. These findings suggest that Kampo formulations, despite being widely perceived as safe, may induce immunologic or inflammatory adverse reactions in susceptible individuals.

This case was considered to represent DIC associated with Yokuinin-to in light of the following findings.

First, the clinical symptoms and findings were consistent with typical features of DIC. Common symptoms include urinary frequency, urgency, dysuria, and bladder pain, often accompanied by hematuria. Urine cultures are typically negative, and sterile pyuria may be present. Imaging findings may include bladder wall thickening or mass-like lesions, and cystoscopic findings often demonstrate erythema and edema of the bladder mucosa [1,9]. The findings in our patient were consistent with these characteristics.

Second, there was a clear temporal relationship between drug administration and symptom development. In the present case, the initial symptoms appeared 20 days after initiation of Yokuinin-to, and gross hematuria developed 4 days after discontinuation following 35 days of treatment. Previous reports have shown that the interval between initiation of Kampo medicine and onset of DIC ranges from 3 weeks to 120 months (median, 24 months), whereas the interval from drug discontinuation to symptom resolution ranges from 3 to 42 days (median, 10 days) [2]. Thus, the clinical course in the present case is compatible with the reported clinical course of DIC.

The precise mechanism underlying the timing of symptom onset relative to discontinuation of the suspected drug remains unclear. Although a delayed immunological reaction or progression of preexisting microscopic mucosal injury may be possible explanations, the exact mechanism could not be determined in the present case.

Third, no medications other than Yokuinin-to showed a temporal association with the patient's symptoms and clinical findings. Although acetaminophen and acetylcysteine hydrochloride were initiated at the same time as Yokuinin-to, these medications were continued for approximately 6 weeks after the patient's symptoms and clinical findings had improved. The resolution of cystitis despite continued administration of these medications, together with improvement after discontinuation of Yokuinin-to, suggests that Yokuinin-to was the most likely causative agent.

Fourth, previous reports have indicated that herbal medicines containing components of Yokuinin-to may cause DIC. Yokuinin-to is composed of multiple herbal ingredients, including Ephedra herb, Glycyrrhiza root, Cinnamon bark, Peony root, Coix seed, Atractylodes rhizome, and Angelica root (Tsumura & Co., package insert, in Japanese). Among these, herbal formulations containing any of the first four components have been reported to be associated with DIC [2].

In addition to these four components, other components with no previous reports of DIC cannot be excluded as potential causes. Therefore, it is not possible to identify the specific causative component in the present case.

Differential diagnoses include eosinophilic cystitis, carcinoma in situ, interstitial cystitis, and viral cystitis. Eosinophilic cystitis could not be definitively excluded because histopathological examination was not performed. However, carcinoma in situ and interstitial cystitis were considered unlikely based on the cystoscopic, cytological, and imaging findings and their spontaneous resolution. Viral cystitis could not be completely excluded because virological testing was not performed. Although these conditions could not be completely excluded, based on the four clinical findings described above, DIC caused by Yokuinin-to was considered the most likely diagnosis.

According to the Naranjo Adverse Drug Reaction Probability Scale [10], Yokuinin-to was classified as a probable cause of the adverse drug reaction (score: 6), based on the temporal relationship between drug initiation and symptom onset, symptom improvement after drug withdrawal, and the lack of a more likely alternative explanation.

Herbal medicines, including Kampo formulations, are often perceived as safe; however, like other medications, they may cause adverse events. In particular, multi-component herbal formulations carry a potential risk of diverse adverse effects because of their multiple constituents.

In cases of nonbacterial cystitis, the possibility of DIC should be considered. As many herbal medicines are available over the counter, it is important to evaluate not only prescribed medications but also self-administered products when assessing patients with unexplained urinary symptoms.

## Conclusions

This case suggests a possible association between Yokuinin-to and cystitis. Careful medication history taking, including the use of herbal products, is essential when evaluating patients with unexplained cystitis.
